# Recent advances in the understanding and management of liposarcoma

**DOI:** 10.12703/r/10-1

**Published:** 2021-01-04

**Authors:** Candace L Haddox, Richard F Riedel

**Affiliations:** 1 Division of Medical Oncology, Duke University Medical Center, Durham, NC, USA; 2 Duke Cancer Institute, Durham, NC, USA

**Keywords:** Liposarcoma, Well-differentiated, Dedifferentiated, Pleomorphic, Myxoid/Round cell, Clinical Trials, Targeted therapy, Immunotherapy

## Abstract

Liposarcomas are a common subfamily of soft tissue sarcoma with several subtypes recognized by the World Health Organization: atypical lipomatous tumors (ALT)/well-differentiated liposarcoma (WDLPS), dedifferentiated liposarcoma (DDLPS), myxoid liposarcoma (MLPS), pleomorphic liposarcoma (PLPS), and myxoid pleomorphic liposarcoma (MPLPS). Despite shared adipocytic features among liposarcomas, the clinical approach to each subtype differs based on histology, location, clinical behavior, and specific oncogenic drivers. In this review, we highlight subtype-specific molecular features with the potential to generate novel therapies. We discuss recent clinical trials investigating the use of preoperative radiation therapy for retroperitoneal liposarcoma, chemotherapy, small molecule inhibitors, and innovative immunotherapy approaches and describe how we incorporate these advancements into the management of liposarcoma.

## Introduction

Soft tissue sarcomas (STS) are rare mesenchymal neoplasms with over 150 different histological subtypes that make up 1% of adult malignancies^1^. Liposarcomas are a common subfamily of adipocytic STS, collectively representing 11.5% of all STS^2^. Liposarcoma subtypes defined by the World Health Organization (WHO) include atypical lipomatous tumors (ALT)/well-differentiated liposarcoma (WDLPS), dedifferentiated liposarcoma (DDLPS), myxoid liposarcoma (MLPS), pleomorphic liposarcoma (PLPS), and the newly described entity, myxoid pleomorphic liposarcoma (MPLPS)^1,3^. Historically, similar treatment approaches have been employed across the range of STS, but accumulating evidence demonstrating distinctive molecular and clinical features of STS subtypes, including liposarcoma, has ushered in an era of histology-tailored treatment approaches^4^.

## Atypical lipomatous tumors/well-differentiated liposarcoma and dedifferentiated liposarcoma

WDLPS and its extremity counterpart, ALT, are low-grade adipocytic tumors often resembling benign lipomas on histopathology^5^. By definition, ALT occurs in the extremities, while WDLPS may arise in the retroperitoneum (RP), paratesticular region, mediastinum, or head and neck region. WDLPS/ALT tend to recur locally rather than metastasize but may dedifferentiate into DDLPS with a more aggressive course and higher rate of metastatic dissemination. The likelihood of recurrence and dedifferentiation is associated with location; less than 7% of ALT dedifferentiate at a median of 7 years, while 17% of RP WDLPS dedifferentiate at a median of 8 years^6,7^. Despite the presence of dedifferentiation, tumors can recur as pure WDLPS, DDLPS, or both.

It is postulated that ALT, WDLPS, and DDLPS arise from a chromosome 12q-shattering chromothriptic event leading to complex genomic rearrangements, formation of ringed chromosomes, and amplification of the 12q13-15 segment^8,9^. Consequently, ALT, WDLPS, and DDLPS share a common cytogenetic feature characterized by supernumerary ringed chromosomes and amplification of specific oncogenes located in the 12q13-15 amplicon, often including *MDM2*, *FRS2*, *CDK4*, *HMGA2, YEATS2,* and *NAV3*^10,11^. During dedifferentiation, ongoing DNA damage leads to genomic instability and further accumulation of complex genomic aberrancies^12,13^. On a gene expression level, pathway analysis of paired WDLPS and DDLPS tumors revealed upregulation of cell proliferation and DNA damage response pathways in DDLPS and upregulation of adipocyte differentiation and metabolic pathways in WDLPS^13^.

### Clinical management

For resectable ALT in the extremity, the prognosis is excellent with low rates of recurrence and dedifferentiation. Given the favorable outcomes with surgery alone, radiation therapy and systemic therapy are not routinely recommended^14^. For localized, extremity DDLPS, stage-directed perioperative radiation therapy and/or chemotherapy could be considered as per NCCN guidelines. While DDLPS tends to have poor chemosensitivity, emerging data suggest that select high-risk patients with extremity/trunk STS and a predicted 10-year overall survival (OS) of 51% or less may have improved outcomes with the use of adjuvant chemotherapy^15^. In such cases, we recommend careful consideration of anticipated risks and benefits by an experienced multidisciplinary sarcoma group.

While WDLPS rarely metastasizes, local recurrence of both RP WDLPS and DDLPS causes morbidity and impacts OS, and thus efforts to improve local control are key. To date, the only completed trial to prospectively evaluate the impact of perioperative radiation therapy compared to surgery alone in RP STS is the STRASS trial (EORTC 62092), a prospective, multicenter phase III randomized controlled trial (RCT)^16^. This trial randomized 266 patients with RP sarcoma (74.5% with liposarcoma, 97% of which were WDLPS/DDLPS) to preoperative radiotherapy followed by surgery or surgery alone and demonstrated a similar 3-year abdominal recurrence-free survival (ARFS) between the two arms (60.4% versus 58.7%, respectively) in the entire study population and in an exploratory post-hoc analysis of patients with liposarcoma.

After reviewing interim results of the STRASS trial in 2017, the Independent Data Monitoring Committee recommended two unplanned sensitivity analyses for ARFS, revealing a non-significant trend favoring preoperative radiation therapy in the subgroup of patients with liposarcoma. Importantly, the trial was not powered to detect differences in the liposarcoma subgroup, and, in fact, power calculations were based on a 20% difference in ARFS at 5 years, rather than 3-year ARFS as reported^16^. Thus, it will be critical to evaluate results of additional planned analyses at later follow up times, particularly with the proclivity of patients with WDLPS to develop late local recurrences^17^. Overall, we feel that the use of radiation therapy remains an individualized decision and should be based on consensus recommendations from an experienced multidisciplinary sarcoma team.

Evidence for the use of preoperative chemotherapy for high-grade RP STS is often extrapolated from studies of extremity and trunk STS, but the planned STRASS 2 trial (EORTC 1809) will directly assess its role in RP STS^18^. This phase III multicenter international trial will randomize 250 patients with high-grade RP dedifferentiated liposarcoma or leiomyosarcoma (stratified based on histology) to receive three cycles of neoadjuvant anthracycline-based chemotherapy followed by surgery or surgery alone, with disease-free survival as the primary outcome. Results for this important trial are not anticipated until 2028^19^. With the absence of definitive data at present, we recommend reserving preoperative chemotherapy for patients with good performance status and borderline resectable or recurrent RP STS where tumor shrinkage may improve surgical outcomes.

In the context of unresectable or metastatic DDLPS, the recommended first-line therapy remains an anthracycline-based chemotherapy regimen. In the phase III trial EORTC 62012, patients with advanced or metastatic STS were randomized to doxorubicin monotherapy or doxorubicin in combination with ifosfamide. While more patients achieved a response (26% versus 14%) and had a longer progression-free survival (PFS, 7.4 versus 4.6 months) with the combination, this was at the cost of higher toxicity and no statistically significant OS benefit^20^. This trial provides a rationale for the continued consideration of anthracycline monotherapy for patients who do not require tumor shrinkage for symptom management. Frontline doxorubicin was also compared to gemcitabine plus docetaxel in a phase III RCT (GeDDiS trial) showing similar survival outcomes but numerically higher quality of life metrics and less toxicity with doxorubicin^21^. As a result, gemcitabine-based combinations with docetaxel, vinorelbine, or dacarbazine are often considered in subsequent lines of therapy^22–24^.

Eribulin is a microtubule inhibitor that was compared to dacarbazine in patients with leiomyosarcoma and liposarcoma in a phase III RCT revealing a significantly improved median OS (13.5 versus 11.5 months)^25^. Subsequently, a planned subgroup analysis revealed the improvement in OS with eribulin was limited to the liposarcoma cohort when compared to dacarbazine (15.6 versus 8.4 months)^26^. A phase III trial also compared trabectedin to dacarbazine in patients with liposarcoma and leiomyosarcoma and demonstrated an improved median PFS benefit (4.2 versus 1.5 months) but no OS advantage^27^. The PFS benefit was observed in both the liposarcoma and the leiomyosarcoma cohorts. Although trabectedin and eribulin have not been directly compared, we typically favor eribulin over trabectedin for DDLPS given the demonstrated OS advantage. As dacarbazine had inferior outcomes when compared to both trabectedin and eribulin, this option can be considered in later lines of therapy.

### Novel therapies

***CDK4/6 inhibition*.** *CDK4* amplification is present in over 90% of WDLPS and DDLPS^11^ and drives the overexpression of *CDK4* and cell cycle progression through dysregulated phosphorylation of retinoblastoma (Rb) protein^28^. CDK4/6 inhibitors induce growth arrest, upregulate the chromatin remodeling enzyme ATRX, and decrease expression of the negative P53 regulator, MDM2, resulting in cell senescence^29^.

Initial clinical studies showed modest activity using the CDK4/6 inhibitor palbociclib but with hematological toxicity leading to a dose reduction in a subsequent phase II nonrandomized trial^30,31^. Treated patients had a median PFS of 4.5 months, and 1 of 30 patients achieved a complete response^31^. Given the lack of randomized data to date, we typically incorporate palbociclib in later lines of therapy.

A phase II study investigating the more potent inhibitor abemaciclib is underway in patients with pretreated or untreated DDLPS, with a preliminary analysis showing a prolonged median PFS of 30.4 weeks^32^. Ribociclib is also in phase II clinical trials in combination with the mTOR inhibitor everolimus^33^ and MDM2 inhibitor HDM201^34,35^, with results pending at this time.

***MDM2 inhibition*.** Although not specific to WDLPS/DDLPS, *MDM2* amplification is seen in nearly 100% of DDLPS^11^ and WDLPS^36^. MDM2 influences sarcomagenesis through ubiquitination and proteasomal degradation of P53 and other proteins such as HBP1, establishing MDM2 as a key player in genomic instability^37^. Early phase clinical trials investigating MDM2 inhibitors in various malignancies with *MDM2* overexpression or amplification showed manageable toxicity and some activity (mainly stable disease and a few partial responses) in WDLPS/DDLPS^38–40^.

***Selective inhibitors of nuclear export*.** Exportin-1 is a nuclear exporter upregulated in liposarcoma that promotes oncogenic behaviors through the extrusion of growth regulatory signals. Inhibition of exportin-1, using the selective inhibitor of nuclear export (SINE) selinexor, decreased cell growth and induced apoptosis in preclinical studies and animal models, suggesting therapeutic potential^41^. Subsequently, a phase Ib clinical trial showed a durable response in nearly half of the DDLPS patients enrolled in the study^42^, leading to the phase II/III SEAL trial in DDLPS^43^. Final results of the trial showed that patients treated with selinexor had a median PFS of 2.8 months versus 2.1 months with placebo, and a small minority achieved a partial response^44^. There was no OS difference between the groups. Common adverse events included GI toxicity, fatigue, weight loss, and cytopenias. While the study met its primary endpoint of PFS, it is unclear whether the outcomes observed with selinexor will be considered clinically meaningful in unselected patients with DDLPS.

***Multikinase inhibitors*.** Tyrosine kinase inhibitors (TKIs) have demonstrated activity in STS and bone sarcomas but have not consistently shown activity in adipocytic sarcomas. A nonrandomized phase II trial (EORTC 62043) investigating pazopanib showed lack of benefit in the liposarcoma subgroup^45^, thus adipocytic sarcomas were excluded from the subsequent phase III study (PALETTE), leading to approval of pazopanib in non-adipocytic STS^46^. Similarly, the phase II RCT REGOSARC also revealed poor activity of the TKI regorafenib in adipocytic STS^47^. Following publication of the PALETTE trial, a single arm, prospective phase II study in intermediate and high-grade liposarcoma showed a 12-week PFS of 68.3%, which renewed interest in TKIs for adipocytic sarcomas^48^. Most recently, however, the multicohort phase II trial SARC024 showed that regorafenib did not have activity in a cohort of patients with liposarcoma^49^. Considering the level of evidence available in these studies, we typically avoid the use of TKIs in adipocytic STS although support clinical trials investigating TKIs in combination regimens or novel TKIs based on sound scientific rationale.

***Immunotherapy*.** The nonrandomized phase II study SARC028 treated patients with advanced soft tissue and bone sarcomas with pembrolizumab monotherapy, showing promising activity in undifferentiated pleiomorphic sarcoma (UPS) and a 20% response rate in patients with DDLPS^50^. This led to two expansion cohorts for UPS and DDLPS, enrolling 40 patients each. Final results suggested less robust activity than anticipated for DDLPS, with an overall response rate of 10% (4 of 39 patients with partial response) and a 12-week PFS of 44%^51^. Correlative analyses from SARC028 investigated the immune microenvironment and revealed that response to PD1 inhibition did not correlate with baseline PDL1 expression but was correlated with a higher baseline density of immune infiltrates, including CD68+ tumor-associated macrophages (TAMs) expressing PDL1 and FOXP3+ T regulatory cells^52^. In another study, publicly available gene expression data from 608 STS specimens were used to categorize tumors into sarcoma immune classes (SIC) A through E based on distinct immune infiltrate, immune function, and immune checkpoint gene expression signatures^53^. Approximately 25% of DDLPS tumors were classified as SIC E, which is characterized by tertiary lymphoid structures containing T cells, follicular dendritic cells, and B cells, and had a higher response to pembrolizumab in SARC028^53^. These correlative studies identify potential opportunities for predictive biomarker development. Two prospective trials currently underway include SARC032^54^, a RCT investigating the role of neoadjuvant pembrolizumab in addition to preoperative radiation therapy and resection for patients with DDLPS and UPS, and a phase II noncomparative randomized trial investigating the effect of neoadjuvant nivolumab and ipilimumab/nivolumab on pathologic response in patients with UPS and RP DDLPS undergoing preoperative radiation therapy and surgery^55^. Preliminary results suggest limited activity in DDLPS^56^. In the metastatic setting, nivolumab and nivolumab/ipilimumab exhibited modest activity with a median PFS of 4.6 and 5.5 months, respectively. Two patients, however, did achieve responses greater than 6 months^57^.

## Myxoid liposarcoma

MLPS represent approximately 20–30% of the liposarcoma subfamily and are defined by the fusion oncogene *FUS-DDIT3* resulting from the genetic translocation t(12:16)(q13:p11) or less frequently *EWSR1-DDIT3* resulting from t(12;22)(q13;q12)^58^*.* MLPS tend to have an unconventional metastatic pattern compared to other STS, with extrapulmonary spread to the abdominal wall, RP, skeletal sites such as the spine^59^, soft tissues, and occasionally serosal sites like the pleura, peritoneum, and pericardium^60^. MLPS may contain a high-grade round cell component (MRCLPS), which confers a higher risk of metastasis.

### Clinical management

The treatment of extremity and trunk MLPS closely aligns with that of WDLPS/DDLPS described above^14^, although MLPS tend to be especially radiosensitive relative to other liposarcomas. As such, deintensified radiation therapy (from 50 to 36 Gy) was evaluated in a phase II nonrandomized single arm phase II trial (DOREMY), revealing that 91% of patients achieved a pathologic response, defined as greater than 50% treatment effect^61^. Recognizing the limitations of a surrogate primary endpoint, the local control rate was impressive, with zero patients experiencing relapse at a median follow up of 25 months. While the sarcoma community engages in discussions regarding this potentially practice-changing trial, registry studies are planned to provide additional insight on radiation therapy dosing. Perioperative chemotherapy remains controversial in STS; however, MLPS may exhibit increased chemosensitivity, and some data suggest a role in high-risk patients. Recently, a phase III trial randomized patients with high-risk extremity or trunk wall STS to receive a standard anthracycline plus ifosfamide neoadjuvant regimen or a histology-tailored regimen followed by limb-sparing surgery^62^. Patients with high-grade MLPS on the histology-tailored arm received trabectedin, an alkylating agent that binds the minor groove of DNA with activity in many fusion-positive sarcoma subtypes^63^. The trial showed similar disease-free survival in the standard and histology-tailored arms, but OS was significantly higher in patients treated with the standard anthracycline-based regimen (5-year OS 65.9 versus 75.7%, *P* = 0.02)^62^. Furthermore, subgroup analysis of the Sarculator nomogram-predicted worst prognostic group demonstrated a nonsignificant trend toward improved disease-free survival and OS in patients receiving anthracycline-based neoadjuvant chemotherapy^15^. Based on these collective results, we will consider the use of perioperative anthracycline-based chemotherapy in select patients with large, high-grade, localized MLPS of the extremity and trunk wall. Currently, a phase II trial is underway exploring multimodality therapy with trabectedin and radiation therapy for patients with localized MLPS, which showed an acceptable safety profile in a phase I trial^64^.

Intraabdominal MLPS is an uncommon primary location^59^ but may be encountered upon metastatic spread to the RP or peritoneum. In cases of oligometastatic spread to the abdominal cavity or RP, it would be reasonable to employ multi-modal approaches using chemotherapy, radiation therapy, and surgery (if operable) given the relative chemosensitivity and radiosensitivity of MLPS.

Overall, MLPS tends to have improved sensitivity to chemotherapy compared to other liposarcoma subtypes^65^. Anthracycline-based regimens remain the first-line systemic therapy in MLPS. Beyond anthracyclines, we favor sequencing trabectedin before eribulin. In the phase III trial comparing eribulin with dacarbazine, an OS benefit was observed in all liposarcoma subtypes except myxoid/round cell liposarcoma^25,26^. Furthermore, biological rationale supports the use of trabectedin in fusion-positive sarcomas^63^. We tend to use dacarbazine and gemcitabine combinations in later lines of therapy.

### Novel therapies

The FUS-DDIT3 fusion oncogene leads to the upregulation of several oncogenic pathways that have directed the investigation of several targeted therapies^66^.

***PPAR-γ agonists*.** PPAR-γ is a master regulator of adipocytic differentiation and apoptosis and is overexpressed in a variety of malignancies where it exhibits antitumor activity^67^. In MLPS, the FUS-DDIT3 fusion drives high expression of PPAR-γ, likely through suppression of signaling downstream of PPAR-γ, and this correlates with poor outcomes in MLPS^67^. The PPAR-γ agonist efatutazone was studied in a phase I trial enrolling patients with advanced malignancies and demonstrated a markedly durable effect in a patient with MLPS^68^, leading to a phase II trial in patients with advanced MLPS^69^.

***PI3K and mTOR inhibitors*.** The growth factor IGF2 binds its receptor, IGF1R, upregulating the PI3K/AKT pathway and mTOR. In MLPS, IGF1R is overexpressed and the PI3K/AKT pathway is upregulated, implicating this pathway in MLPS oncogenic behaviors and progression^66,70^. Furthermore, activating *PIK3CA* mutations are present in up to 18% of MLPS, providing a rationale for several targeted therapies, including mTOR inhibitors^70,71^ and PI3K inhibitors^72^.

***Multikinase inhibitors*.** FUS-DDIT3 activates a variety of kinases, suggesting a potential role for multikinase inhibitors in the treatment of MLPS; however, studies have failed to demonstrate activity of TKIs in MLPS. Most recently, this was demonstrated in the multicohort phase II RCT SARC024, which showed no benefit of regorafenib in a cohort of patients with liposarcoma^49^. Although this trial was not powered to detect activity in the MLPS subgroup, it is notable that patients with MLPS made up 25% of the liposarcoma cohort, yet there was no evidence of activity^49^.

***Immunotherapy*.** Several genes that are expressed during development become restricted to the testis in adulthood and may be aberrantly expressed in tumors (so-called cancer testis antigens, CTA). These antigens represent promising targets for engineering tumor immune responses given the immune-privileged environment of the testis. NY-ESO-1 is a highly immunogenic cancer testis antigen that is expressed in 90% of MLPS^12^. A phase I/II pilot study administered autologous specific peptide enhanced affinity receptor (SPEAR) T cells recognizing an NY-ESO-1 peptide complexed with HLA-A*02 to patients with MRCLS and specific HLA-A*02 haplotypes. In a preliminary analysis, engineered T cells administered following lymphodepleting chemotherapy resulted in two partial responses^73,74^. Similarly, MAGEA4 is a CTA expressed in 67.7% of MLPS^75^, and a phase II trial is underway investigating SPEAR T-cells targeting MAGEA4 in patients with MLPS and synovial sarcoma^76,77^. Dendritic cell vaccines are another method to prime tumor-targeting T cells. The dendritic cell vaccine CMB305 contains a dendritic cell-targeting lentivirus encoding NY-ESO-1, a TLR-4 agonist, and recombinant NY-ESO-1 peptide. Dendritic cell expression of NY-ESO-1 results in presentation to cytotoxic T cells and expansion of anti-NY-ESO-1 T and B cells. A phase II RCT of CMB305 in combination with atezolizumab versus atezolizumab alone showed limited activity in both arms^78^. These innovative immunotherapy approaches are in early stages but have the potential to expand therapeutic options for patients with MLPS.

## Pleomorphic liposarcoma

PLPS is an aggressive liposarcoma subtype that accounts for about 5% of liposarcomas. Over 80% of patients with PLPS present over age 50, and the most common primary tumor site is the extremity (65%), followed by the RP and abdomen (15%)^79^. Five-year and 10-year OS are 57% and 39%, respectively, with higher survival rates in patients with superficial tumors and lower survival in patients with RP and abdominal primary tumors^79,80^. In the setting of unresectable or metastatic disease, median OS is 14 months^81^. Unlike the subtypes described above, PLPS has a complex genome without characteristic genomic alterations or targetable molecular drivers. Common genomic alterations include mutations in *TP53* (17%), *NF1* (8%), *RB1* (4%), *PIK3CA* (4%), *SYK* (4%), *PTK2B* (4%), *EPHA5* (4%), and *ERBB4* (4%)^82^, and deletions of *TP53*, *RB1*, and *NF1* are also common^12^. Interestingly, the histological features and genomic alterations observed in PLPS are similar to those of myxofibrosarcoma, potentially representing a spectrum of the same disease^82,83^.

The cornerstone of treatment for patients with high-risk localized PLPS is complete surgical resection when feasible and radiation therapy per NCCN guidelines for STS. In the metastatic setting, we typically use the agents described above for WDLPS/DDLPS, which have demonstrated moderate activity in PLPS^81^. Notably, in the phase III trial comparing eribulin with dacarbazine, eribulin demonstrated an improvement in OS (22.2 versus 6.7 months) and a non-significant trend toward improved PFS (4.4 versus 1.4 months) in the PLPS subgroup^26^. Unfortunately, studies on targeted therapies and novel approaches are lacking for PLPS, and this represents an area of considerable need.

## Myxoid pleomorphic liposarcoma

MPLPS is a rare, emerging subtype of the liposarcoma subfamily that was first described in 2009^84^ and was first recognized by the WHO classification of soft tissue and bone tumors in 2020^1^. The clinical course and molecular features of MPLPS distinguish it from both MLPS and PLPS. MPLPS typically presents in children, adolescents, and young adults as a deep soft tissue mass primarily located in the mediastinum, extremity, head and neck, abdominal cavity, or trunk. This entity exhibits a blend of MLPS and PLPS histological findings with high-grade features and a complex genome without the *FUS-DDIT3* oncogene fusion that is characteristic of MLPS^58^. Additionally, comparative genomic hybridization comparing PLPS and MPLPS revealed that MPLPS lacks the copy number gains and amplifications seen in PLPS^85^, supporting its recognition as a distinct entity. At present, there are no consensus recommendations for standard of care with respect to local and systemic therapies.

## Conclusion

Liposarcomas are a diverse and heterogeneous group of STS, despite their common adipocytic features. Novel therapies targeting a myriad of pathways and known drivers of pathogenesis are actively being explored. A continued understanding and appreciation of the subtype-specific biological underpinnings is essential in optimizing treatment approaches and improving outcomes for patients.
